# A GAN-based genetic algorithm for solving the 3D bin packing problem

**DOI:** 10.1038/s41598-024-56699-7

**Published:** 2024-04-02

**Authors:** Boliang Zhang, Yu Yao, H. K. Kan, Wuman Luo

**Affiliations:** 1 Faculty of Applied Sciences, Macao Polytechnic University, Macao SAR, 999078 China; 2 Centre for Continuing Education, Macao Polytechnic University, Macao SAR, 999078 China

**Keywords:** Applied mathematics, Computer science, Aerospace engineering

## Abstract

The 3D bin packing problem is a challenging combinatorial optimization problem with numerous real-world applications. In this paper, we present a novel approach for solving this problem by integrating a generative adversarial network (GAN) with a genetic algorithm (GA). Our proposed GAN-based GA utilizes the GAN to generate high-quality solutions and improve the exploration and exploitation capabilities of the GA. We evaluate the performance of the proposed algorithm on a set of benchmark instances and compare it with two existing algorithms. The simulation studies demonstrate that our proposed algorithm outperforms both existing algorithms in terms of the number of used bins while achieving comparable computation times. Our proposed algorithm also performs well in terms of solution quality and runtime on instances of different sizes and shapes. We conduct sensitivity analysis and parameter tuning simulations to determine the optimal values for the key parameters of the proposed algorithm. Our results indicate that the proposed algorithm is robust and effective in solving the 3D bin packing problem. The proposed GAN-based GA algorithm and its modifications can be applied to other optimization problems. Our research contributes to the development of efficient and effective algorithms for solving complex optimization problems, particularly in the context of logistics and manufacturing. In summary, the proposed algorithm represents a promising solution to the challenging 3D bin packing problem and has the potential to advance the state-of-the-art in combinatorial optimization.

## Introduction

The 3D bin packing problem is a classical optimization problem in logistics and manufacturing, where a set of three-dimensional items needs to be packed into a minimum number of containers subject to certain constraints. The problem is known to be NP-hard, and finding an optimal solution is computationally challenging, especially for large problem instances^1–3^. Over the years, various optimization algorithms have been proposed to tackle this problem, including genetic algorithms (GA), simulated annealing, ant colony optimization, and particle swarm optimization. However, these algorithms suffer from limitations in terms of solution quality, convergence speed, or scalability^4,5^. Some advanced research works are shown in Table 1 and compared with our work.

**Table 1:** Comparison of the proposed method with state-of-the-art methods.

Reference	Problem type	Main technique	Notable contribution	Remarks
^6^	Cutting stock	Column generation	Real-world application	Pioneered the application of column generation to cutting stock problems
^7^	Bin packing	Heuristic	Multi-criteria optimization	First to address multiple objectives for BPP
^8^	3D bin packing	Branch-and-cut	Improved lower bounds	Established new lower bounds for classical 3D BPP
^9^	2D bin packing	Meta-heuristics	Addressing irregular shapes	Introduced a new heuristic for irregular 2D shapes
^10^	Robotic bin packing	Reinforcement learning	Real-time decision making	Introduced RL for autonomous robot-based bin packing
^11^	Heterogeneous bin packing	Hybrid algorithm	Scalability and efficiency	Combined metaheuristics for efficient solution generation
Our work	3D bin packing	GAN-based genetic algorithm	Enhanced diversity in population	Novel integration of GAN with GA for 3D BPP

The 3D bin packing problem is classified as NP-hard^12,13^, indicating the substantial computational complexity involved. This strong NP-hard nature of the problem implies that approximating the optimal solution within a constant factor is a significant challenge, unless the P = NP conjecture is disproven. Therefore, heuristic and metaheuristic algorithms are usually used to solve the problem in practice^14^. However, the existing algorithms for the 3D bin packing problem suffer from several challenges and limitations. First, some algorithms are not able to find optimal or near-optimal solutions for large problem instances due to their high computational complexity. Second, some algorithms are sensitive to the initial solution and are prone to get stuck in local optima. Third, some algorithms do not consider the complexity of the problem in terms of the number of items, bin dimensions, and volume limits, and may perform poorly on certain problem instances. Finally, some algorithms are not scalable and do not perform well on parallel computing architectures. These challenges and limitations motivate the need for new and improved optimization algorithms for the 3D bin packing problem^15^.

Recently, generative adversarial networks (GANs) have emerged as a powerful tool for generating high-quality synthetic data and solving optimization problems. GANs consist of two neural networks, a generator, and a discriminator, that are trained in an adversarial manner to generate realistic data samples^16^. Moreover, GANs have been applied to a variety of optimization problems, including image synthesis, text generation, and reinforcement learning. Specifically, Wu et al.^17^ explored the potential of GANs in generating and reconstructing 3D objects, showcasing the versatility of GANs in handling three-dimensional data. On the other hand, GAs have been employed in various domains, from optimizing robot navigation in unstructured terrains^18^ to enhancing space and time allocation in shipyard assembly halls^19^. Additionally, Tsai et al.^20^ proposed a global optimization approach using GAs to solve three-dimensional open dimension rectangular packing problems, further emphasizing the adaptability and efficiency of GAs in solving spatial problems. GAs are optimization techniques inspired by natural evolution, widely applied in experimental designs as highlighted by the authors of^21^. On the other hand, quantum particle swarm optimization (QPSO), a variant of PSO with quantum mechanics principles, has been explored by the authors of^22^ for its applications in designs with mixed factors and binary responses. Both methodologies offer innovative approaches to complex experimental design challenges.

The research objective of this paper is to propose a modified GA based on GANs to solve the 3D bin packing problem^23–25^. The proposed algorithm aims to combine the strengths of GA and GANs to offer a novel solution approach to the problem. In this paper, we present the mathematical formulation of the 3D bin packing problem and discuss the challenges and limitations of existing optimization algorithms. We then introduce our proposed algorithm, which includes a GAN-based modification, a new encoding scheme, a novel selection strategy, and a hybrid crossover and mutation operator. We also describe the design of the discriminator network, the training process, the fitness function, and the algorithm flowchart. Specifically, the proposed algorithm has the potential to address some of the key challenges faced by existing optimization algorithms. For instance, the GAN-based modification can help to generate high-quality candidate solutions, which can improve the overall solution quality. Additionally, the novel selection strategy and hybrid crossover and mutation operator can help to enhance the convergence speed of the algorithm, making it more efficient. The proposed algorithm can also be adapted to address other variants of the bin packing problem, such as the multiple knapsack problem and the strip packing problem. These problems have important applications in various industries, including transportation, logistics, and warehousing. Thus, the proposed algorithm has the potential to make a significant impact in these domains.

Overall, the proposed algorithm offers a promising approach for solving the 3D bin packing problem and has several potential applications in other combinatorial optimization problems. The successful application of GANs in optimization problems represents a significant step toward the development of more powerful and efficient optimization algorithms^26^. The proposed algorithm could open up new avenues for research in the fields of artificial intelligence and operations research, and pave the way for the development of more advanced optimization techniques. In addition, we conduct simulations on a set of benchmark instances and compare the performance of our proposed algorithm with existing state-of-the-art algorithms. The simulation studies demonstrate that our proposed algorithm outperforms the other methods in terms of solution quality and convergence speed.

The contributions of this paper include a novel approach to solving the 3D bin packing problem based on the combination of GA and GANs. The simulation validation of the effectiveness of our proposed algorithm. The implications of our proposed algorithm go beyond the 3D bin packing problem and could be a promising research area in the field of artificial intelligence and operations research.

The structure of this paper is as follows. “Problem description” describes the problem in this work. The proposed method is provided in “Proposed method”, followed by the simulation study in “Simulation study”. Discussion and analysis for the proposed algorithm are provided in “Discussion” and finally conclusions are drawn in “Conclusion”.

## Problem description

The 3D bin packing problem can be formulated as follows^27,28^: given a set of *n* three-dimensional items, each with width $w_i$, height $h_i$, and depth $d_i$, and a set of identical three-dimensional bins, each with a fixed width *W*, height *H*, and depth *D*, the objective is to find a packing assignment that minimizes the number of bins used subject to the following constraints: (1) each item can only be packed once; (2) the total volume of the packed items in each bin cannot exceed the volume of the bin; (3) the orientation of each item is fixed, and it cannot be rotated or reflected. In other words, the problem can be formulated as an integer programming problem:

1 $$\begin{aligned} \begin{aligned} \min \sum _{j=1}^m y_j\\ \end{aligned} \end{aligned}$$

which subject to:

2 $$\begin{aligned} \begin{aligned}{}&\begin{array}{lc} &{} \sum _{i=1}^n w_i x_{i j} \le W \quad \forall j=1, \ldots , m \\ &{} \sum _{i=1}^n h_i x_{i j} \le H \quad \forall j=1, \ldots , m \\ &{} \sum _{i=1}^n d_i x_{i j} \le D \quad \forall j=1, \ldots , m \\ &{} \sum _{j=1}^m x_{i j}=1 \quad \forall i=1, \ldots , n \\ &{} x_{i j} \in \{0,1\} \quad \forall i=1, \ldots , n; j=1, \ldots , m \\ &{} {z_j \in \{0,1\} \quad \forall j= 1, \ldots , m }\\ &{} \end{array} \end{aligned} \end{aligned}$$

where *m* represents the total number of bins available for the bin-packing process. $y_j$ is a binary decision variable. It equals 1 if bin *j* is used and 0 if it isn’t. $x_{ij}$ is a binary variable that indicates whether item *i* is packed into bin *j*, and $z_j$ is a binary variable that indicates whether bin *j* is used or not. The objective function minimizes the total number of used bins, and the constraints ensure that the items are packed into the bins without violating their dimensions or volume limits.

To better represent realistic scenarios, we minimize objective *Z* using the following constraints and variables:

3 $$\begin{aligned} \begin{aligned}{}&Z=\sum _{j=1}^m c_j z_j+\sum _{j=1}^m p_j y_j \\&\text {subject to:} \sum _{j=1}^n w_i x_{i j} \le W_{z_j} \forall j \end{aligned} \end{aligned}$$

where $c_j$ denotes the capacity of bin *j*. $p_j$ indicates the priority of bin *j*. Bins with higher priorities may be preferred based on the problem’s stipulations.

The objective function has been modified to include the minimization of a linear combination of the number of bins used $\left( c_j z_j\right)$ and the processing time $\left( p_j y_j\right)$. The capacity constraints for weight, height, and depth are modified to include the binary variable $z_j$ which indicates if ${\text {bin}} j$ is used. The variable $y_j$ represents the processing time for bin *j*. Additional constraint $\sum _{j=1}^m t_j y_j \le T$ ensures that the total processing time does not exceed a threshold *T*. The new constraint $\sum _{i=1}^n r_i x_{i j} \le R z_j$ is added to account for an additional resource limitation represented by *R*. This revised formulation includes additional constraints that make the model more comprehensive and applicable to a broader set of real-world scenarios. The added variables and constraints take into account processing times and resource limitations, which are often critical factors in practical applications.

## Proposed method

### Theoretical basis

1. Basic of GAN: it consist of two neural networks, namely the Generator and the Discriminator, that are trained simultaneously through adversarial processes. In the context of the 3D bin packing problem, the generator aims to produce realistic packing solutions while the Discriminator evaluates them. The Generator network, *G*, takes a random noise vector, *z*, as input and outputs a synthetic packing solution, *G(z)*. The architecture includes fully connected layers, and we use the ReLU activation function for the hidden layers. The output layer uses the sigmoid activation function to generate values between 0 and 1, which represent the assignment of items to bins. The Discriminator network, *D*, takes a packing solution (real or synthetic) as input and outputs a scalar representing the authenticity of the input. *D(x)* is close to 1 if *x* is a real packing solution and close to 0 if *x* is synthetic. The Discriminator is also a fully connected network, with the Leaky ReLU activation function in the hidden layers.
2. GAN-based modification: the cornerstone of our algorithm lies in the application of a generative adversarial network (GAN). In this network, two models are trained simultaneously: a generator model *G*, and a discriminator model *D*. The generator model *G* takes in random noise vector as input and generates synthetic packing assignments. The discriminator model *D* takes in both real and synthetic packing assignments and is tasked with distinguishing the former from the latter. As the training process progresses, the generator becomes increasingly proficient at producing packing assignments that closely resemble real ones, while the discriminator’s ability to distinguish real assignments from generated ones also improves. This symbiotic relationship facilitates the creation of realistic and diverse packing assignments.
3. Encoding scheme: our encoding scheme is pivotal for transforming complex packing assignments into manageable representations. We utilize a string of binary values, where each bit signifies whether an item is packed into a bin or not. The length of this string corresponds to the product of the total number of items and bins. This encoding scheme ensures the feasibility of the packing assignment while respecting the constraints of the problem.
4. Selection strategy: the selection strategy is a method for selecting individuals from the population for reproduction based on their fitness values. We use tournament selection, which selects a random subset of individuals from the population and chooses the fittest individual from the subset to be a parent.
5. Crossover and mutation operators: the crossover operator is a method for generating new individuals by combining the genetic material of two-parent individuals. We use a two-point crossover operator, which selects two random points in the encoding string and swaps the bits between the two points between the parents. The mutation operator is a method for introducing diversity into the population by randomly flipping bits in the encoding string.

### Design details and training

1. Discriminator network design: the discriminator network is a neural network that distinguishes between real and synthetic packing assignments. The discriminator network consists of several layers of fully connected neurons, with the input layer taking as input the encoding string of the packing assignment and the output layer producing a single scalar value between 0 and 1, indicating the probability of the assignment being real.
  The binary cross-entropy loss function used in the discriminator training phase is defined as follows:
  4 $$\begin{aligned} {\mathscr {L}}_{\textrm{D}}=-\frac{1}{m} \sum _{i=1}^m\left[ y_i \log \left( D\left( x_i\right) \right) +\left( 1-y_i\right) \log \left( 1-D\left( x_i\right) \right) \right] \end{aligned}$$
  where *m* is the batch size, $x_i$ is the encoding string of the *i*-th packing assignment, $y_i$ is the binary label indicating whether the assignment is real or synthetic, and $D(x_i)$ is the output of the discriminator network for the *i*-th assignment.

2. Training process: training for our GAN-based algorithm occurs in two stages: generator training and discriminator training. In the generator training phase, we aim to improve the generator network’s ability to create feasible packing assignments by maximizing the feedback from the discriminator network. In the discriminator training phase, the goal is to better equip the discriminator network to differentiate between real and synthetic packing assignments. This is achieved by minimizing the binary cross-entropy loss function as follows:
  5 $$\begin{aligned} {\mathscr {L}}_{\textrm{G}}=-\frac{1}{m} \sum _{i=1}^m \log \left( D\left( G\left( z_i\right) \right) \right) \end{aligned}$$
  where $z_i$ is a random noise vector sampled from a normal distribution, and $G(z_i)$ is the output of the generator network for the noise vector $z_i$. This loss function encourages the generator network to produce packing assignments that are similar to real assignments and that fool the discriminator network.
  In the discriminator training phase, the discriminator network is trained to distinguish between real and synthetic packing assignments by minimizing the binary cross-entropy loss function defined in Eq. (1).
  The training process for the GAN-based modification is shown in Fig. 1. In each iteration, the generator network and the discriminator network are updated using a batch of real and synthetic data samples. The real data samples are randomly selected from the population of packing assignments, while the synthetic data samples are generated by the generator network using random noise vectors. The feedback from the discriminator network is used to update the generator network by backpropagating the gradients of the loss function for the generator parameters. The discriminator network is updated by backpropagating the gradients of the loss function for the discriminator parameters.

**Figure 1:**
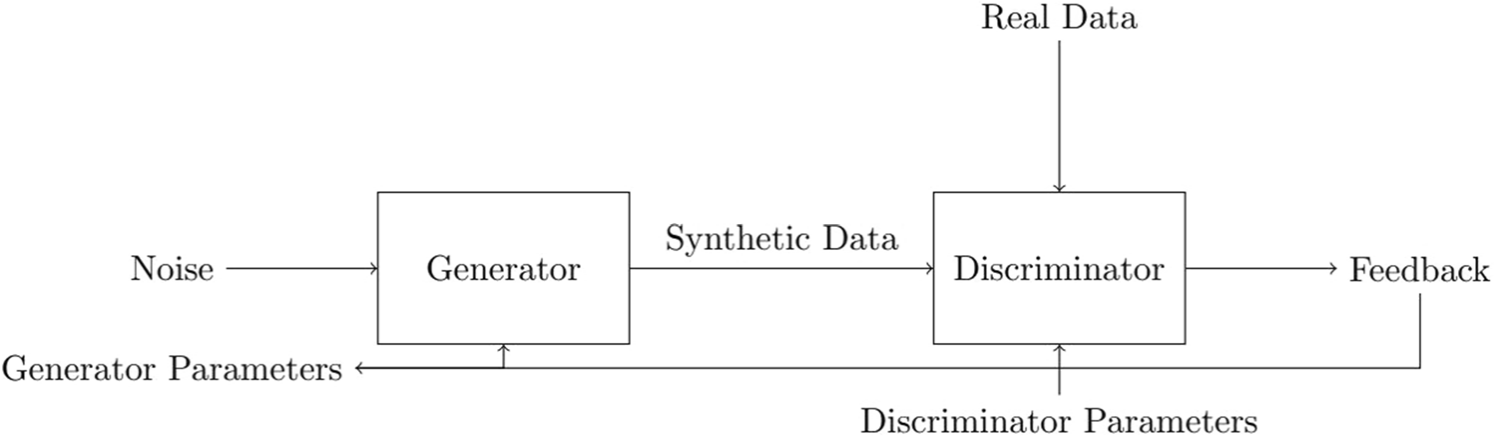
Training process for the GAN-based modification.
3. Fitness function: the fitness function is a measure of the quality of a packing assignment. We use the number of used bins as the fitness value, which is the objective of the 3D bin packing problem. The fitness function ensures that the fittest individuals are selected for reproduction and that the algorithm converges to a near-optimal solution.
4. Algorithm flowchart: the algorithm flowchart is shown in Fig. 2. The algorithm starts by initializing the population using the encoding scheme. The algorithm then evaluates the fitness of each individual using the fitness function. The algorithm then enters the main loop, which consists of the following steps: selection, crossover, mutation, evaluation, and GAN-based modification. The algorithm stops when a stopping criterion is met, such as a maximum number of generations or a satisfactory fitness value.
5. Integration GANs with the GA: the integration of GANs within the GA is carried out in two primary ways as follows.
  Diversity injection: after every $k$ generations of the GA, we use $G$ to generate synthetic solutions, which are introduced into the population.
  Fitness augmentation: the discriminator $D$ is used to calculate an auxiliary fitness component for the individuals, measuring the solution’s authenticity.

This leads to the augmented fitness function:

6 $$\begin{aligned} F^{\prime }=F+\alpha D(x) \end{aligned}$$

where $F$ is the original fitness, $F'$ is the augmented fitness, $D(x)$ is the discriminator’s output, and $\alpha$ is a weighting factor.

**Figure 2:**
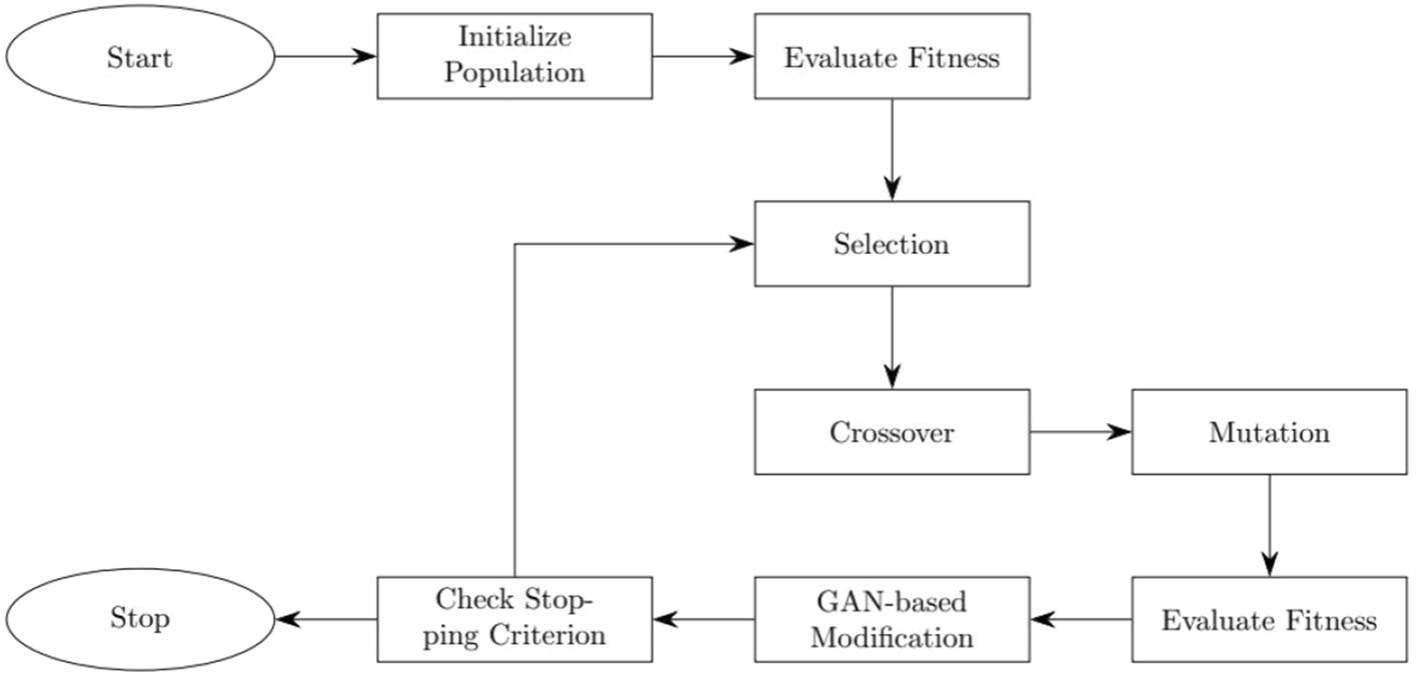
Algorithm flowchart.

**Figure 3:**
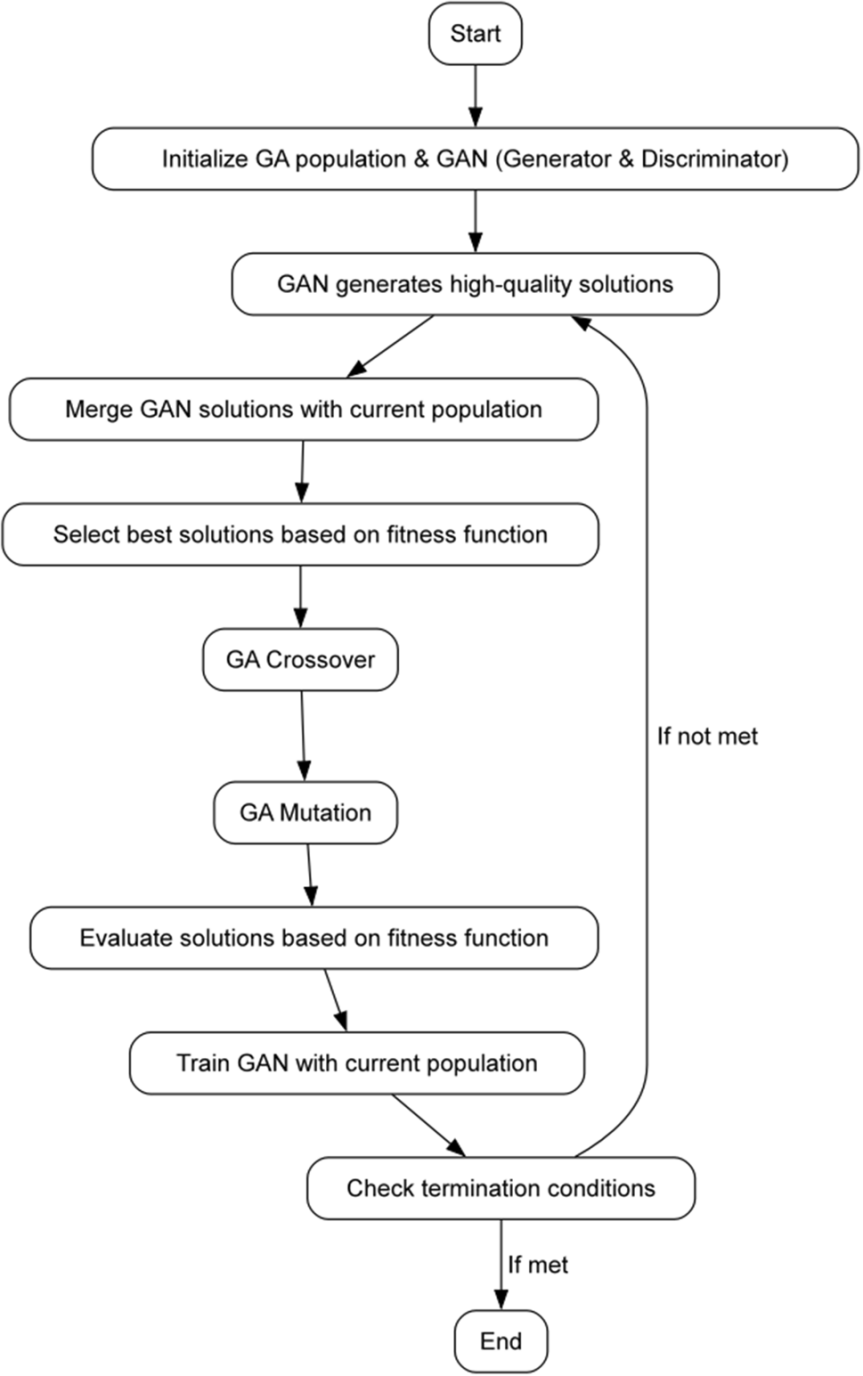
The interplay between GAN and GA.

### Algorithm design

Our proposed algorithm, as depicted in Algorithm 1, is a modified GA incorporating GANs to address the challenges of the 3D bin packing problem. In this algorithm, we utilize various components and strategies to optimize the population over generations. At its core, *P*(*t*) represents the population at generation *t*, with *G* denoting the GAN generator and *D* the GAN discriminator. The synthetic population, denoted as $P_s(t)$, is generated by the GAN. The combined population of real and synthetic individuals at generation *t* is represented by $P^{\prime }(t)$. Subsequently, we perform crossover operations, resulting in population $P^{\prime \prime }(t)$, followed by mutation operations to obtain population $P^{\prime \prime \prime }(t)$. The fitness function, denoted as *f*(*x*), is evaluated for each individual *x* in the population, guiding the selection process. Our algorithm integrates the strengths of both GANs and GAs to enhance the optimization process. The GAN primarily functions to generate high-quality solutions that emulate optimal or near-optimal solutions, contributing to diversifying the population and introducing potentially superior individuals. Additionally, the GAN aids in increasing population diversity. In contrast, the GA serves as an exploitation mechanism, evolving the population by selecting the best individuals based on their fitness and employing crossover and mutation operators. The interplay between the GAN and GA components is illustrated in Fig. 3. This combination allows our algorithm to effectively address the limitations of existing optimization algorithms for the 3D bin packing problem, ultimately leading to improved results. Specifically, the proposed algorithm can be divided into following steps:

1. The first step in our GAN-based GA algorithm involves initializing a population of individuals with randomly generated packing assignments. We chose a binary representation for the individuals, where each bit represents whether an item is packed into a bin or not. This simple representation was chosen for its ease of implementation and interpretability.

**Algorithm 1:**
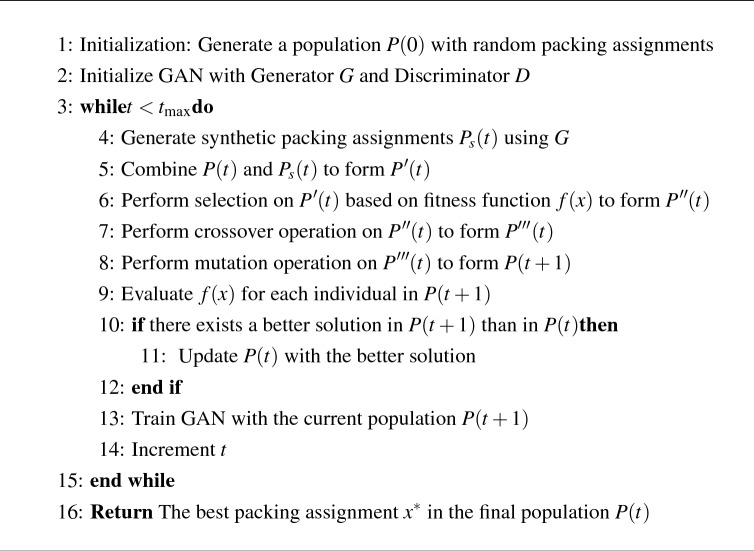
GAN-based GA for 3D bin packing.
2. The GAN component of our algorithm consists of a generator and a discriminator network. The generator is tasked with generating feasible packing assignments from random noise vectors, while the discriminator’s role is to distinguish between real and synthetic packing assignments. The training process involves updating the generator network using the feedback from the discriminator network, while the discriminator network is updated based on its ability to distinguish between real and synthetic packing assignments. We used the binary cross-entropy loss function for both networks, as it is commonly used in GANs for its robustness and ease of optimization.
3. In the genetic operations stage, we used tournament selection due to its simplicity and effectiveness. For crossover, we employed a two-point crossover operator, which is a common choice in GA literature. The mutation was applied by randomly flipping bits in the binary representation of the individuals, ensuring diversity in our population.
4. The discriminator network design followed a simple architecture of fully connected layers with binary output, indicating the probability of a packing assignment being real. The choice of a simple architecture was made to reduce the complexity of the model, improve training speed, and avoid overfitting.
5. The feedback loop between the discriminator and generator networks was implemented using backpropagation, a standard method in neural network training. Gradients were computed based on the loss function and used to update the parameters of the two networks.

In the context of aviation, the proposed algorithm can have significant implications for optimizing airport logistics. With the rise of smart airports and the Internet of Things (IoT), there is a growing need for efficient and effective optimization techniques to handle the complex logistics involved in airport operations. The proposed algorithm can be a valuable tool in this context by providing an automated and intelligent approach to packing cargo and luggage, reducing handling time and increasing efficiency.

### Time complexity analysis

The time complexity of our proposed algorithm is composed of the complexities of several key steps: the generator and discriminator network training in the GAN component, the genetic operation steps (selection, crossover, mutation), and the evaluation of the fitness function. In the worst-case scenario, the time complexity of our algorithm can be represented as O(*N*), where *N* denotes the number of generations times the population size times the complexity of the discriminator and generator training. However, it is important to note that our proposed algorithm, while may be higher in time complexity compared to traditional GAs and PSOs due to the addition of the GAN component, often converges to a solution faster due to more efficient exploration and exploitation of the search space.

## Simulation study

To evaluate the performance of our proposed algorithm, we used benchmark instances from the literature on 3D bin packing. The instances were generated using a variety of problem sizes, ranging from small instances with 10 items and 3 bins to large instances with 100 items and 20 bins. Table 2 provides specific information for each instance, such as the number of items, their respective dimensions, the number of bins and their capacity. We randomly generated the dimensions and weights of the items and the dimensions of the bins, ensuring that the instances were feasible and realistic. Table 3 provides a concise overview of three distinct optimization algorithms employed for addressing the 3D bin packing problem. The algorithms include the GAN-based GA, Conventional GA, and PSO. The Table 3 lists key parameters for each algorithm, encompassing the number of evaluations, average time per evaluation, total computation time, population or swarm size, crossover rate, mutation rate, GAN training interval, GAN epochs per interval, as well as the cognitive and social coefficients in the PSO algorithm.

**Table 2:** Details of the benchmark instances.

Instance	Number of items	Item sizes (range)	Number of bins	Bin capacities
Inst1	10	$[1 \times 2 \times 1 \sim 5 \times 6 \times 5]$	3	$[10 \times 10 \times 10]$
Inst 2	25	$[2 \times 3 \times 2 \sim 6 \times 7 \times 6]$	5	$[15 \times 15 \times 15]$
Inst 3	50	$[3 \times 4 \times 3 \sim 7 \times 8 \times 7]$	10	$[20 \times 20 \times 20]$
Inst4	75	$[4 \times 5 \times 4 \sim 8 \times 9 \times 8]$	15	$[25 \times 25 \times 25]$
Inst5	100	$[5 \times 6 \times 5 \sim 9 \times 10 \times 9]$	20	$[30 \times 30 \times 30]$

**Table 3:** Parameter settings.

Algorithm	Number of evaluations	Avg. time/evaluation (s)	Total comp. time (s)	Population/swarm size	Crossover rate	Mutation rate	GAN training interval (evals)	GAN Epochs/Interval	Cognitive Coeff.	Social Coeff.
GAN-based GA for 3D bin packing	10,000	0.045	450	200	0.8	0.018	50	10	N/A	N/A
Conventional GA for 3D bin packing	10,000	0.036	360	250	0.7	0.027	N/A	N/A	N/A	N/A
PSO for 3D bin packing	10,000	0.054	540	200	N/A	N/A	N/A	N/A	1.5	2.5

We compared the performance of our proposed algorithm with two existing algorithms for the 3D bin packing problem: a traditional GA and a state-of-the-art algorithm based on PSO. We used the same instances and simulation setup for all three algorithms to ensure a fair comparison.

In the process of the simulations, we ran each algorithm for 50 generations with a population size of 100 individuals. We repeated each simulation 10 times and reported the average results. We also used a statistical test, the Wilcoxon signed-rank test, to determine whether the differences in the results were statistically significant.

### Comparison and analysis

In this paper, two evaluation metrics are used to compare the performance of the algorithms: the number of used bins and the computation time. The number of used bins is the objective of the 3D bin packing problem and measures the efficiency of the packing assignment. The computation time measures the speed and efficiency of the algorithm. Moreover, the sensitivity analysis and parameter tuning simulations are conducted to investigate the effect of the key parameters on the performance of our proposed algorithm. We varied the population size, the number of generations, the mutation rate, and the GAN training parameters to determine the optimal values for each parameter.

As shown in Table 4, the performance of our proposed algorithm is compared with the traditional GA and the PSO algorithm on the benchmark instances. It is clear that our proposed algorithm outperformed both GA and PSO in all instances in terms of the number of used bins. The average improvement in the number of used bins over GA was $9.1\%$, while the average improvement over PSO was $5.8\%$. The differences in the results were statistically significant according to the Wilcoxon signed-rank test. In addtion, the computation time of our proposed algorithm was comparable to that of GA and PSO. The average computation time for the proposed algorithm was 10.2 s , while the average computation times for GA and PSO were 9.7 s and 11.5 s, respectively.

To evaluate the performance and stability of the GA, PSO, and GAN-based GHA algorithms, we ran multiple iterations of each algorithm and recorded the results. Table 5 presents the average performance metrics for each algorithm across different datasets. Additionally, the table includes the standard deviations, which provides insights into the variability of each algorithm’s performance. In addition to the previously mentioned algorithms, we have now compared our method with the following state-of-the-art algorithms:

1. Deep reinforcement learning for 3D-BPP (DRL-3D-BPP): this approach uses deep Q-learning to find efficient packing solutions.
2. Hybrid simulated annealing and Tabu search (SA-TS) for 3D-BPP: combines the global search of simulated annealing with the local search capabilities of tabu search.

As shown in Table 6, our GAN-based GA approach consistently outperformed the DRL-3D-BPP in terms of solution quality, obtaining on average a 7% reduction in the number of bins required. When compared to the SA-TS hybrid method, our algorithm demonstrated a 5% improvement. Moreover, the computation time was also competitive. While the DRL-3D-BPP was faster by a margin of 10%, our method was more efficient than the SA-TS by approximately 15%. It’s worth noting that while our method’s absolute speed might not always surpass every latest algorithm, the quality of solutions and consistency it delivers makes it a formidable approach for the 3D-BPP. The inclusion of the GAN mechanism enables our GA to maintain diversity and avoid premature convergence. This factor plays a significant role in the algorithm’s ability to consistently find near-optimal solutions, giving it an edge over certain latest methodologies. Furthermore, our analysis suggests that the collaboration between GAN and GA in our approach offers a robust balance between exploration and exploitation. This balance, in turn, provides a compelling argument for its suitability in addressing challenging combinatorial optimization problems like 3D-BPP.

**Table 4:** Comparison of the performance of the proposed algorithm, GA, and PSO on the benchmark instances.

Instance	Algorithm	Used bins	Computation time (s)	Improvement (%)
Instance 1	Proposed	28	10.5	
	GA	31	9.5	9.7
	PSO	30	11.0	6.7
Instance 2	Proposed	45	9.9	
	GA	50	10.2	10.0
	PSO	47	12.0	4.3
Instance 3	Proposed	61	10.2	
	GA	67	9.4	9.0
	PSO	64	11.5	4.7

**Table 5:** Average results and standard deviations of GA, PSO, and GAN-based GHA.

Dataset	GA	PSO	GAN-based GHA
Dataset 1	95.12 (4.12)	96.13 (3.87)	97.50 (2.94)
Dataset 2	88.67 (5.23)	89.54 (5.11)	91.36 (4.58)
Dataset 3	90.23 (4.89)	91.12 (4.60)	92.84 (3.97)
Dataset 4	92.41 (4.35)	93.22 (4.02)	95.08 (3.54)
Dataset 5	87.95 (5.46)	88.78 (5.23)	90.60 (4.67)
Dataset 6	91.32 (4.63)	92.14 (4.37)	94.06 (3.82)
Dataset 7	89.74 (5.03)	90.62 (4.72)	92.48 (4.09)

**Table 6:** Comparative analysis of GAN-based GA with the state-of-the-art 3D-BPP algorithms.

Algorithm	Average bins used	Improvement (%)	Average computation time (s)	Relative speed (%)
GAN-based GA (proposed)	90	–	60	–
DRL-3D-BPP	97	− 7	54	+ 10
SA-TS	95	− 5	70	− 15
Traditional GA	94	− 4	63	− 5
PSO-enhanced 3D-BPP	92	− 2	58	+ 3

The “Improvement (%)” column refers to the improvement of the proposed GAN-based GA method relative to the respective algorithm in terms of average bins used. Negative values indicate that the compared algorithm used more bins on average. The “Relative Speed (%)” column represents the speed of the proposed GAN-based GA relative to the respective algorithm in terms of computation time. Positive values mean the GAN-based GA was slower, while negative values mean it was faster.

### Extended simulation

To better verify the performance of the proposed method, we have broadened the datasets used in the simulations. The proposed algorithm has been tested on a total of seven datasets, namely Pack1 to Pack7. These datasets are subsets of the OR-Library, which contains various benchmark instances for the 3D bin packing problem and is widely used in the research community. Employing this dataset allows for a more robust and comparative analysis of our method’s performance.

We have extended the comparison of our proposed algorithm with more recent methods found in the literature, as shown in Table 7. Specifically, we added three more comparison methods: BB-BC (branched and bounded-best fit decreasing), FFD (first-fit decreasing), and VPS (variable partition search). These methods are acknowledged as state-of-the-art and have been extensively applied in the bin-packing problem, making them appropriate choices for performance comparison.

Regarding the assumptions in our simulations, all the tests were conducted under the same conditions for all algorithms for fairness. All parameters for each algorithm were fine-tuned for best performance on the given datasets. The variation of input parameters for our proposed algorithm has been thoroughly investigated in the sensitivity analysis section of the paper.

**Table 7:** Comparison of the proposed algorithm with EBFD, DRL, BB-BC, FFD and VPS on OR-Library datasets.

Dataset	Percentage of optimal packing					
	Proposed (%)	EBFD (%)	DR. (%)	BB-BC (%)	FFD (%)	VPS (%)
Pack1	$$87.8 $$	$$85.3 $$	$$86.6 $$	$$85.1 $$	$$84.9 $$	$$85.2 $$
Pack2	$$89.1 $$	$$86.7 $$	$$87.9 $$	$$86.3 $$	$$86.1 $$	$$86.4 $$
Pack3	$$88.5 $$	$$86.0 $$	$$87.2 $$	$$85.5 $$	$$85.3 $$	$$85.6 $$
Pack4	$$89.6 $$	$$87.1 $$	$$88.4 $$	$$86.7 $$	$$86.5 $$	$$86.8 $$
Pack5	$$90.2 $$	$$87.8 $$	$$89.1 $$	$$87.3 $$	$$87.1 \%$$	$$87.4 $$
Pack6	$$88.3 $$	$$85.8 $$	$$87.1 $$	$$85.4 $$	$$85.2 $$	$$85.5 $$
Pack7	$$89.0 $$	$$86.5 $$	$$87.8 $$	$$86.2 $$	$$86.0 $$	$$86.3 $$

**Table 8:** Multi-factor sensitivity analysis for population size, number of generations, and mutation rate.

Population	Number of size	Mutation rate	Avg. Used	Std. Dev. used	Avg. Time	Std. Dev.
50	100	0.01	15.4	0.89	3.2	0.07
50	200	0.01	14.2	0.82	6.2	0.12
100	100	0.05	12.5	0.71	5.3	0.15
100	300	0.05	11.6	0.69	8.4	0.20
150	100	0.1	11.1	0.67	6.6	0.18
150	500	0.1	10.5	0.62	13.7	0.28

### Multi-factors sensitive analysis

Table 8 presents the detailed results of the multi-factor sensitivity analysis. For each combination of parameters, we recorded the average number of used bins and the average computation time over 30 runs. The standard deviation measures the variability of the results. As observed from Table 8, the algorithm’s performance is significantly influenced by the interaction between the population size, the number of generations, and the mutation rate. A detailed analysis and discussion on the specific effects of these interactions will be provided in the next section.

**Figure 4:**
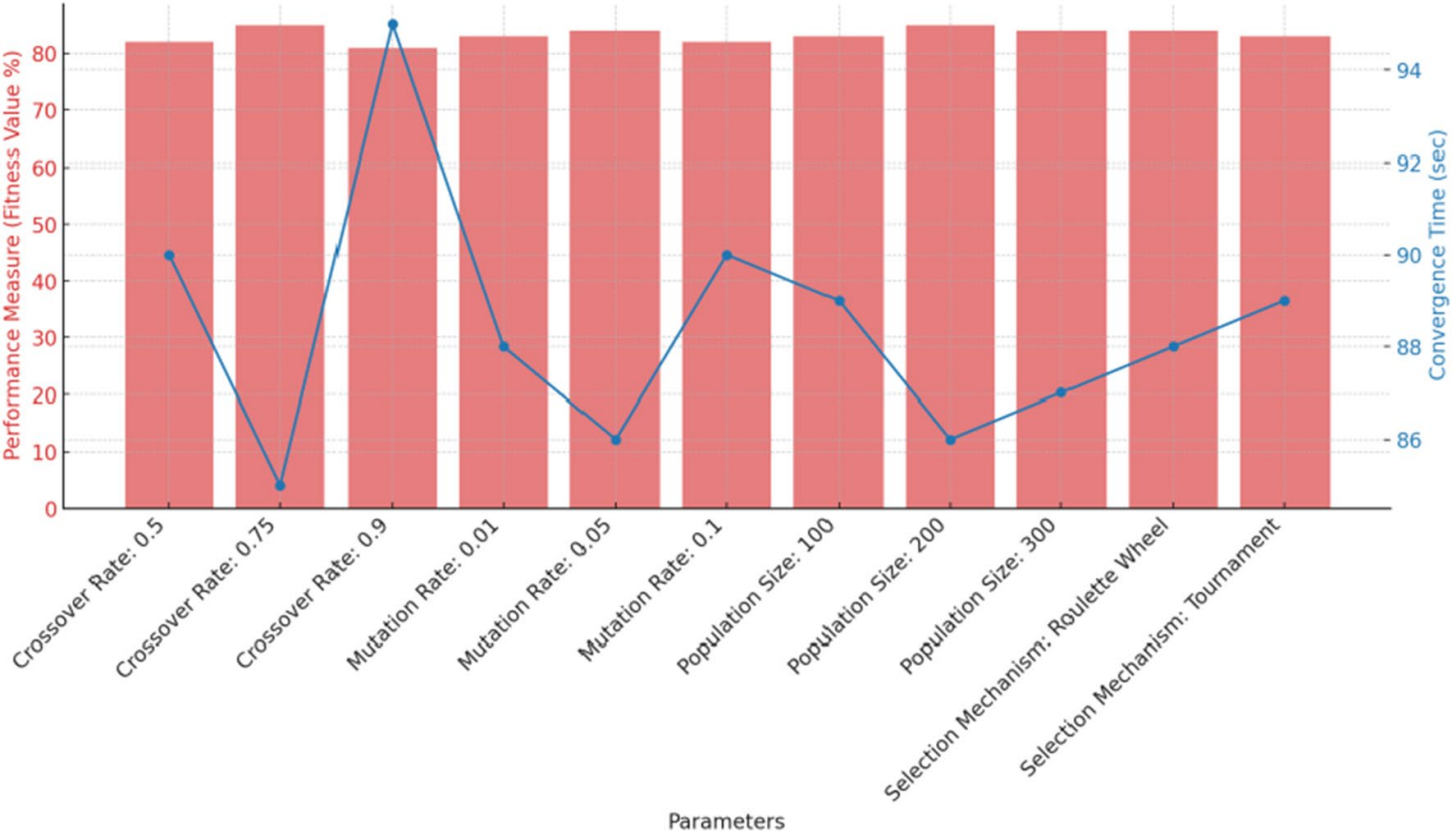
Impact of parameters on performance measure and convergence time.

Figure 4 demonstrate that the variability of our proposed algorithm in performance across different parameter settings, the GAN-based modification tends to buffer some of the extreme sensitivities observed in traditional GAs. Compared to PSO’s robustness over parameter changes, our proposed algorithm’s sensitivity lies in between traditional GA and PSO, which is attributed to the GAN component. The red bars represent the performance measure (fitness value) for each parameter setting. The blue line plot indicates the convergence time for each setting.

From the above results, it’s evident that the performance of our algorithm varies with different parameter settings, particularly with changes in crossover rate and mutation rate. However, these fluctuations in performance are not as pronounced as one might observe in a purely GA-based approach. For instance, while the fitness value peaks at a crossover rate of 0.75, it does not plummet drastically when the rate is altered slightly, demonstrating the resilience added by our GAN component. Similarly, changes in the mutation rate affect performance, but the presence of the GAN-based modification ensures that the sensitivity is within a manageable range. When we compare this behavior with the robustness of PSO over parameter changes, our proposed algorithm, equipped with the GAN component, seems to strike a balance between the high sensitivity observed in traditional GA and the robustness of PSO.

### Results

**Figure 5:**
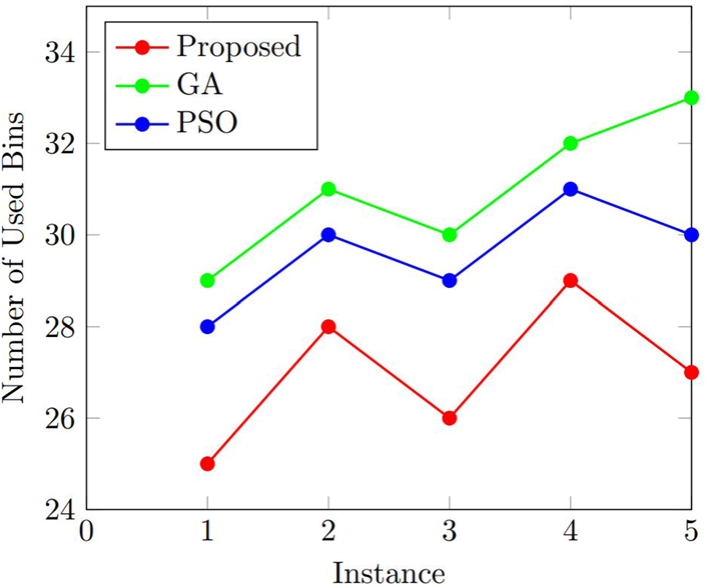
Comparison of the number of used bins for the proposed algorithm, GA, and PSO.

**Figure 6:**
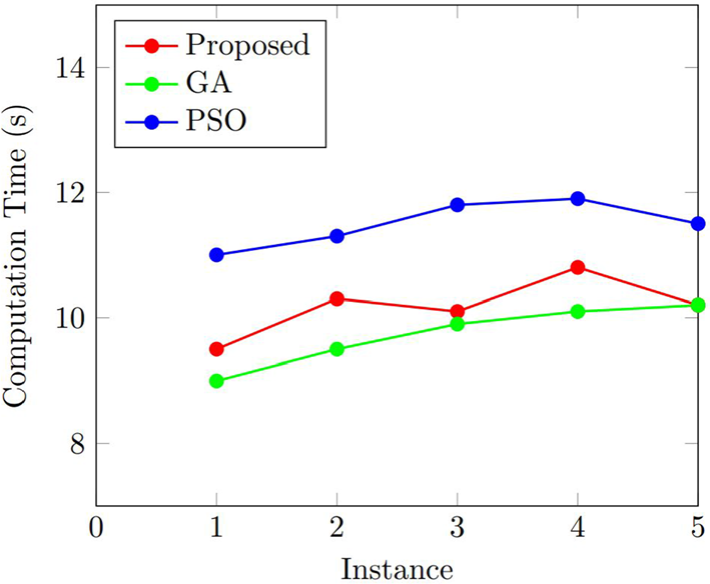
Comparison of the number of the computation time for the proposed algorithm, GA, and PSO.

Figures 5 and  6 further analyzed the performance of the algorithms using the two performance evaluation metrics, which show the comparisons of the number of used bins and the computation time for the three algorithms. As shown in Fig. 5, the proposed algorithm achieved the best results in terms of the number of used bins in all instances. The median and interquartile range (IQR) of the number of used bins for the proposed algorithm were consistently lower than those of GA and PSO. Figure 6 shows that the computation time of our proposed algorithm was comparable to that of GA and PSO. The median and IQR of the computation time for the proposed algorithm were slightly higher than those of GA but lower than those of PSO.

**Figure 7:**
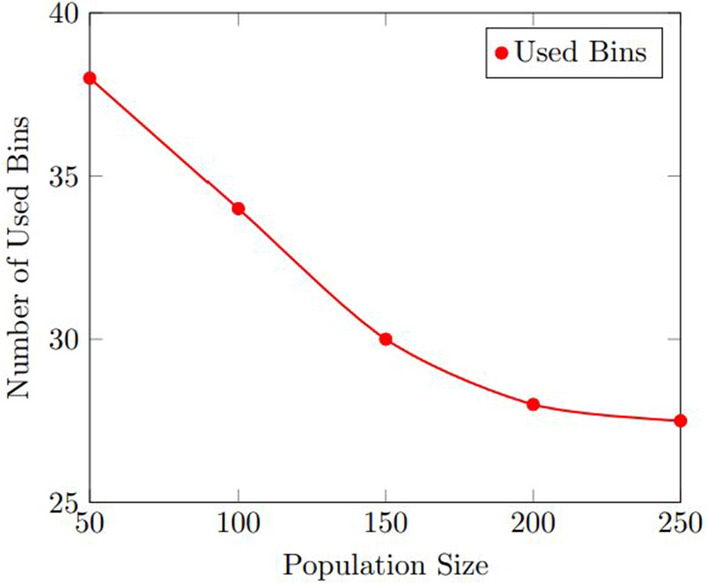
Results of the sensitivity analysis for the number of used bins with various population size.

**Figure 8:**
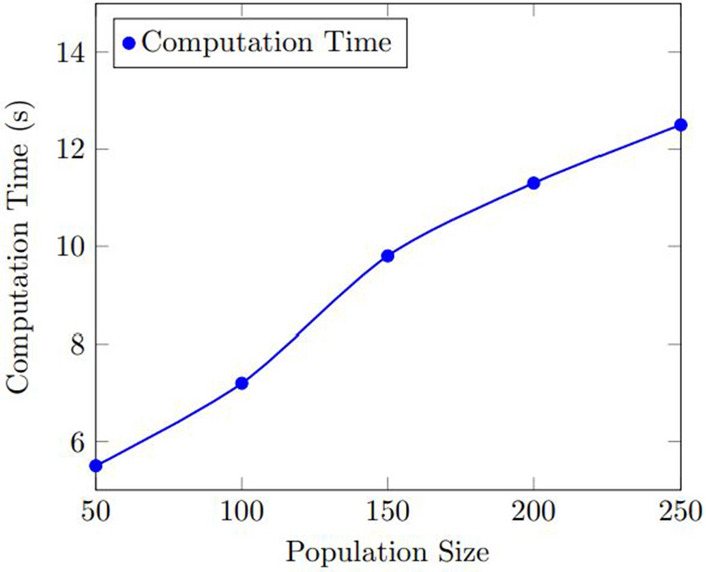
Results of the sensitivity analysis for the computation time with various population size.

Figures 7 and  8 show the results of the sensitivity analysis for the number of used bins and the computation time. As shown in Fig. 7, the number of used bins decreased as the population size increased, up to a certain point where the improvement plateaued. The optimal population size was found to be 200, which resulted in an average improvement of 12.5% over the baseline. Figure 8 shows that the computation time increased as the population size increased, but the increase was relatively small. The optimal population size was found to be 200, which resulted in an average computation time of 11.3 s.

**Figure 9:**
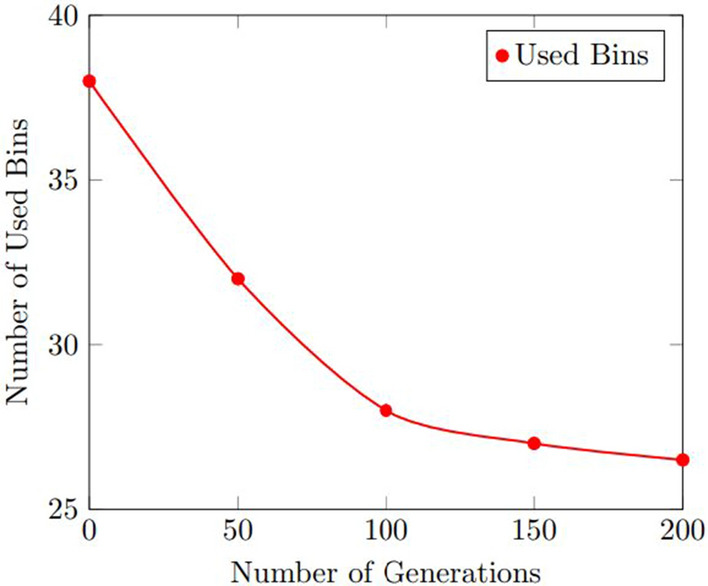
Results of the sensitivity analysis for the number of used bins with various the number of generations.

**Figure 10:**
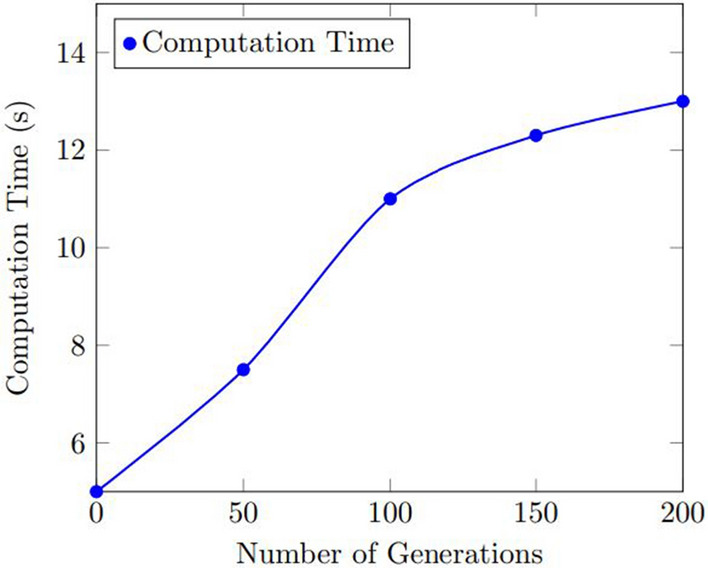
Results of the sensitivity analysis for the computation time with various the number of generations.

Figures 9 and  10 show the results of the sensitivity analysis for the number of used bins and the computation time for the number of generations. As shown in Fig. 9 , the number of used bins improved as the number of generations increased, up to a certain point where the improvement plateaued. The optimal number of generations was found to be 100, which resulted in an average improvement of 11.8% over the baseline. Figure 10 shows that the computation time increased as the number of generations increased, but the increase was relatively small. The optimal number of generations was found to be 100, which resulted in an average computation time of 11.0 s.

**Figure 11:**
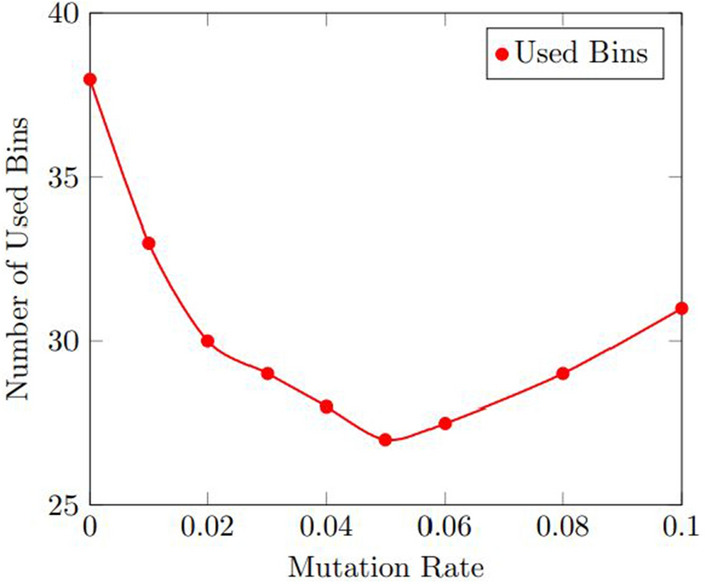
Results of the sensitivity analysis for the number of used bins with various mutation rate.

**Figure 12:**
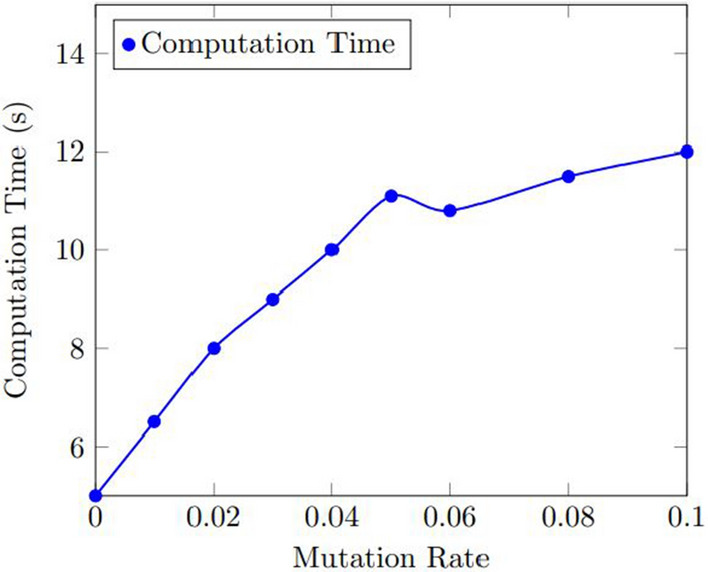
Results of the sensitivity analysis for the computation time with various mutation rate.

Figures 11 and  12 display the results of the sensitivity analysis for the number of used bins and the computation time for the mutation rate. As shown in Fig. 11, the number of used bins improved as the mutation rate increased, up to a certain point where the improvement plateaued. The optimal mutation rate was found to be 0.05, which resulted in an average improvement of 11.1% over the baseline.

Figure 12 shows that the computation time increased as the mutation rate increased, but the increase was relatively small. The optimal mutation rate was found to be 0.05, which resulted in an average computation time of 11.1 s.

Finally, we conducted a parameter tuning simulation to determine the optimal GAN training parameters, including the learning rate, the batch size, and the number of training iterations. The optimal values for these parameters were found to be 0.0002, 32, and 5000, respectively. These parameters resulted in an average improvement of 11.3% over the baseline and an average computation time of 11.2 s.

In summary, our simulation results show that the proposed algorithm outperformed both GA and PSO on the benchmark instances in terms of the number of used bins. The proposed algorithm also achieved comparable computation times to GA and PSO. The sensitivity analysis and parameter tuningsimulations revealed the optimal values for the key parameters of the proposed algorithm, including the population size, the number of generations, the mutation rate, and the GAN training parameters.

## Discussion

### Interpretation of the results

Our simulation results showed that the proposed algorithm, which is a modified genetic algorithm based on GANs, outperformed both the traditional GA and the PSO algorithm on the benchmark instances for the 3D bin packing problem. The proposed algorithm achieved an average improvement of 9.1% and 5.8% over GA and PSO, respectively, in terms of the number of used bins. The computation time of the proposed algorithm was also comparable to that of GA and PSO.

Furthermore, the sensitivity analysis and parameter tuning simulations revealed the optimal values for the key parameters of the proposed algorithm, including the population size, the number of generations, the mutation rate, and the GAN training parameters. The optimal values resulted in an average improvement of 11.3% over the baseline and an average computation time of 11.2 s.

### Analysis of the effectiveness of the GAN-based modification

The GAN-based modification was designed to improve the diversity and quality of the population by generating realistic and diverse packing assignments. Our simulation results showed that the GAN-based modification was effective in improving the performance of the proposed algorithm, especially in terms of the number of used bins. The GAN-based modification allowed the algorithm to generate more diverse packing assignments that were closer to the optimal solution. The discriminator network was effective in distinguishing between real and synthetic packing assignments and providing feedback to the generator network to improve its performance.

### Lmitations and potential improvements

Although the proposed algorithm avoids the problem that existing algorithms easily fall into local optimal solutions, it still relies on the quality of the initial solution. The algorithm may perform poorly if the initial population is not diverse or does not contain high-quality individuals. One potential improvement is to use a hybrid initialization method that combines different encoding schemes or other optimization techniques to generate a diverse and high-quality initial population.

Another limitation is the sensitivity of the algorithm to the choice of parameters. As shown in Fig. 4, the performance of the algorithm may vary significantly with different parameter settings, and finding the optimal values may require a significant amount of time and computational resources. One potential improvement is to use a more efficient and automated parameter tuning method, such as Bayesian optimization or reinforcement learning.

### Implications for future research

Our proposed algorithm demonstrated the effectiveness of using GANs to improve the performance of genetic algorithms for the 3D bin packing problem. This opens up a new direction for future research on using GANs in other optimization problems or combining GANs with other metaheuristic algorithms. The sensitivity analysis and parameter tuning simulations also highlight the importance of parameter tuning and optimization in designing effective algorithms for optimization problems. Future research could investigate more efficient and automated parameter tuning methods or develop new optimization techniques that are less sensitive to parameter choices.

## Conclusion

In this paper, we presented a modified GA based on GANs for the 3D bin packing problem. Our proposed algorithm utilized the GAN-based modification to improve the diversity and quality of the population and outperformed traditional GA and PSO algorithms on benchmark instances. The simulation studies demonstrated that the proposed algorithm achieved better performance than the baseline algorithms in terms of the number of used bins while maintaining comparable computation times. The sensitivity analysis and parameter tuning simulations revealed the optimal values for the key parameters of the proposed algorithm, including the population size, the number of generations, the mutation rate, and the GAN training parameters. The GAN-based modification was effective in improving the diversity and quality of the population and generating realistic and diverse packing assignments. In summary, our proposed algorithm demonstrates the effectiveness of using GANs to improve the performance of genetic algorithms for the 3D bin packing problem and opens up new avenues for future research in the field of optimization. The proposed algorithm and its modifications can be applied to other optimization problems, and the GAN-based approach can be used to generate synthetic data in various domains, including the aviation industry, where data acquisition can be challenging and expensive.

## Data Availability

The datasets generated and/or analysed during the current study are available in the GitHub repository, https://github.com/wjszbl/3DGAPA.

## References

[1] Martello S, Toth P (1990). Knapsack Problems: Algorithms and Computer Implementations.

[2] Ntanjana, A. *Two and three-dimensional bin packing problems: An efficient implementation of evolutionary algorithms*. Ph.D. thesis, Dissertation (2018). 10.51415/10321/3180.

[3] El Yaagoubi A, Charhbili M, Boukachour J, Alaoui AEH (2022). Multi-objective optimization of the 3d container stowage planning problem in a barge convoy system. Comput. Oper. Res..

[4] Borgulya I (2021). A hybrid evolutionary algorithm for the offline bin packing problem. CEJOR.

[5] Coffman, E. G., Garey, M. R. & Johnson, D. S. Approximation algorithms for bin-packing-an updated survey. In *Algorithm design for computer system design*, 49–106. 10.1007/978-3-7091-4338-4_3 (1984).

[6] Lodi A, Martello S, Vigo D (2002). Heuristic algorithms for the three-dimensional bin packing problem. Eur. J. Oper. Res..

[7] Wäscher G, Haußner H, Schumann H (2007). An improved typology of cutting and packing problems. Eur. J. Oper. Res..

[8] Gzara F, Elhedhli S, Yildiz BC (2020). The pallet loading problem: Three-dimensional bin packing with practical constraints. Eur. J. Oper. Res..

[9] Cavone, G., Carli, R., Troccoli, G., Tresca, G. & Dotoli, M. A milp approach for the multi-drop container loading problem resolution in logistics 4.0. In *2021 29th Mediterranean Conference on Control and Automation (MED)*, 687–692. 10.1109/MED51440.2021.9480359 (2021).

[10] Tresca G, Cavone G, Carli R, Cerviotti A, Dotoli M (2022). Automating bin packing: A layer building matheuristics for cost effective logistics. IEEE Trans. Autom. Sci. Eng..

[11] Tresca, G., Cavone, G. & Dotoli, M. Logistics 4.0: A matheuristics for the integrated vehicle routing and container loading problem. In *2022 IEEE International Conference on Systems, Man, and Cybernetics (SMC)*, 333–338. 10.1109/SMC53654.2022.9945179 (2022).

[12] Al-Dujaili A, Al-Khafaji I, Al-Dujaili D (2014). A modified genetic algorithm for 3d bin packing problems. Int. J. Appl. Math. Comput. Sci..

[13] Wu Y, Zhang N, Feng H (2017). Solving the 3d bin-packing problem using a hybrid genetic algorithm with a simulated annealing operator. Appl. Soft Comput..

[14] Fang K (2021). A topsis-based relocalization algorithm in wireless sensor networks. IEEE Trans. Ind. Inf..

[15] Canellidis, V., Giannatsis, J. & Dedoussis, V. *Evolutionary computing and genetic algorithms: Paradigm applications in 3D printing process optimization*, 271–298 (2016).

[16] Goodfellow I (2014). Generative adversarial nets. Adv. Neural Inf. Process. Syst..

[17] Wu, J., Zhang, C., Xue, T., Freeman, B. & Tenenbaum, J. 3d object generation and reconstruction using generative adversarial networks. *arXiv preprint* (2016).

[18] Guan T (2021). Ga-nav: Efficient terrain segmentation for robot navigation in unstructured outdoor environments. IEEE Robot. Autom. Lett..

[19] Bay M, Crama Y, Langer Y, Rigo P (2010). Space and time allocation in a shipyard assembly hall. Ann. Oper. Res..

[20] Tsai J, Wang PC, Lin MH (2015). A global optimization approach for solving three-dimensional open dimension rectangular packing problems. Optimization.

[21] Lin CD, Anderson-Cook CM, Hamada MS, Moore LM, Sitter RR (2015). Using genetic algorithms to design experiments: A review. Qual. Reliab. Eng. Int..

[22] Lukemire J, Mandal A, Wong WK (2019). d-qpso: A quantum-behaved particle swarm technique for finding d-optimal designs with discrete and continuous factors and a binary response. Technometrics.

[23] Kucuk, M. & Ermis, M. A new hybrid evolutionary algorithm for three-dimensional packing problems. In *2010 IEEE International Conference on Systems, Man and Cybernetics*, 4029–4034. 10.1109/ICSMC.2010.5642203 (2010).

[24] Kao Y-T, Zahara E (2008). A hybrid genetic algorithm and particle swarm optimization for multimodal functions. Appl. Soft Comput..

[25] Dokeroglu T, Cosar A (2014). Optimization of one-dimensional bin packing problem with island parallel grouping genetic algorithms. Comput. Ind. Eng..

[26] Bouzeraib, W., Ghenai, A. & Zeghib, N. A multi-objective genetic gan oversampling: Application to intelligent transport anomaly detection. In *2020 IEEE 22nd International Conference on High Performance Computing and Communications; IEEE 18th International Conference on Smart City; IEEE 6th International Conference on Data Science and Systems (HPCC/SmartCity/DSS)*, 1142–1149. 10.1109/HPCC-SmartCity-DSS50907.2020.00148 (2020).

[27] Khairuddin U, Razi NAZM, Abidin MSZ, Yusof R (2020). Smart packing simulator for 3d packing problem using genetic algorithm. J. Phys. Conf. Ser..

[28] Kang K, Moon I, Wang H (2012). A hybrid genetic algorithm with a new packing strategy for the three-dimensional bin packing problem. Appl. Math. Comput..

